# Factors Influencing the Prevalence of Mental Health Problems among Malay Elderly Residing in a Rural Community: A Cross-Sectional Study

**DOI:** 10.1371/journal.pone.0156937

**Published:** 2016-06-09

**Authors:** M. Rizal Abdul Manaf, Madihah Mustafa, Mohd Rizam Abdul Rahman, Khairul Hazdi Yusof, Noor Azah Abd Aziz

**Affiliations:** 1 Department of Community Health, Faculty of Medicine, Universiti Kebangsaan Malaysia Medical Centre, Jalan Yaacob Latiff, Bandar Tun Razak, 56000 Cheras, Kuala Lumpur, Malaysia; 2 Department of Family Medicine, Faculty of Medicine, Universiti Kebangsaan Malaysia Medical Centre, Jalan Yaacob Latiff, Bandar Tun Razak, 56000 Cheras, Kuala Lumpur, Malaysia; Cardiff University, UNITED KINGDOM

## Abstract

**Background and Purpose:**

Mental health problems are common in old age, but frequently remain undetected and untreated. Mental health problems in the elderly are the result of a complex interaction of social, psychological and biological factors. The aim of this study is to determine the prevalence of mental health problems (depression, anxiety, and emotional stress) and their associated factors among the Malay elderly in a rural community of Perak, Malaysia.

**Methods:**

It was a cross-sectional study. The Malay elderly aged 60 years and above were selected through convenient sampling to give a total of 230 respondents. The Depression, Anxiety, and Stress Scale (DASS-21) was used to assess the symptoms of depression, anxiety, and stress. Bivariate analyses were performed using chi-square tests and multiple logistic regression analyses were conducted to determine the association between the factors and each of the mental health statuses assessed.

**Results:**

The results showed that the prevalence of depression, anxiety, and stress among the elderly respondents was 27.8%, 22.6%, and 8.7%, respectively. The significant factors for depression were single elderly (Adjusted OR = 3.27, 95%CI 1.66, 6.44), living with family (Adjusted OR = 4.98, 95%CI 2.05, 12.10), and poor general health status (Adjusted OR = 2.28, 95%CI 1.20, 4.36). Living with family was the only significant factor for anxiety (Adjusted OR = 2.68, 95%CI 1.09, 6.57). There was no significant factor for stress.

**Conclusions:**

Depression and anxiety among the Malay elderly in the rural community were very worrying. More equity in health should be created or strengthened in order to intensify the opportunity to identify, diagnose, and treat those with mental health problems. Living arrangement in the rural community was an important factor that had influenced depression and anxiety. Therefore, further research is recommended for more comprehensive information, as a result of which appropriate intervention can be made.

## Introduction

The problem of aging is becoming a global phenomenon both in developed and developing countries. The aging population has increased rapidly over the last decades owing to two significant factors, namely, the reduction in mortality and fertility rates and improved quality of life, leading to an increase in life expectancy worldwide [1–3]. The World Health Organization (WHO) has estimated that the proportion of the world’s elderly people over the age of 60 will be doubled from 11to 22% between the year 2000 and 2050 [4]. Similarly to Malaysia, as reported in the Malaysia Health Facts [5–6], the life expectancy at birth has increased from 71.6 years to 72.5 years for male, and from 76.5 years to 77.2 years for female (between the year 2009 and 2014). In Malaysia alone, it is estimated that by the year 2020, almost 10% of Malaysia’s population or 3.4 million people will be 60 years and above [7].

The elderly, in general, face various challenges that are associated with physical and psychological changes commonly associated with the aging process. Although many attributed the changes as normal and acceptable among elderly, there are certain aspects in mental health that are pathological and need to be recognized and treated early, especially those leading to emotional instability and overt depression [8]. Interestingly mental health problems in the elderly are the result of a complex interaction of social, psychological, and biological factors. The elderly are more likely to experience events such as bereavement, a drop in socio-economic status owing to retirement, inability to work because of progressive disability, and loss of social roles and network. Studies have shown that about 15% of elderly had limited mobility which required assistance, 30% had suffered with cognitive impairment and over 50% had chronic physical illnesses [9–10]. Later, this may lead to a double impact in the sequalae, of losing their ability to live well mentally as well as losing their independence in life.

Literature has documented that approximately over 20% of the elderly in the world, aged 60 years and above, suffer from mental disorders, with the most common psychiatric disorders being depression and anxiety [11]. Looking into the economic impact, mental health disorders are attributed to 6.6% of all disabilities (Disability Adjusted Life Years or DALYs) in this age group [11], that require some form of long-term care as well as utilization of treatment and support services.

The challenge for health practitioners is to screen for those who need intervention, as mental illnesses among the elderly are often silent and masked as aging occurs. As for the elderly themselves, the main challenge is to overcome the social stigma surrounding mental health illnesses, resulting in the reluctance among the elderly to seek help for their problems. Mental health illnesses if left untreated may induce functional disability, disturbed rehabilitation, burden to the health care system, and impaired quality of life of the elderly people and their families.

To date, there is little known regarding the spectrum of mental health problems among the elderly people in Malaysia, especially those in the rural community. Most local studies conducted earlier have focused on a single mental health problem, most commonly depression, among elderly people. Therefore, realizing this breach in investigation, we have conducted this study with the aim of investigating the prevalence of the overall mental health problems, which include depression, anxiety, and emotional stress among the Malay elderly in a rural community of Malaysia. It is hoped that this preliminary data will help to determine the burden of mental health problems among the elderly in the rural community, especially the factors contributing to these problems.

## Materials and Methods

### Location

This was a cross-sectional study conducted in Teluk Intan, Perak, between March and May 2013. Teluk Intan is a town in the Hilir Perak District, 171 km from Kuala Lumpur (the capital city of Malaysia), which is situated in one of the northern states of peninsular Malaysia. The national statistics of 2010 reported that the total population of Teluk Intan consisted of 41,701 residents, with a majority of its population being Chinese (53.9%) followed by Malay (29.1%), Indian (16.6%), and others (0.4%). A breakdown of the age demographic showed that the elderly population comprised of 6,275 (15%) residents;34.5% comprised of the elderly, aged 60 to 64 years;23.6% formed the 65 to 69 years age group; 20.1% were of aged 70 to 74 years; and 21.8% belonged to the above 75 years group [7].

### Sample

Two villages with a relatively higher number of Malay elderly were selected through purposive sampling as these two villages are located nearby the study office. A non-probability technique (convenient sampling) was used to recruit the Malay elderly (aged 60 years and above) from the selected villages. This technique was used as there is no specific list of name and address of the elderly residing in the two villages. Those who were able to communicate with the investigators in a reasonable manner, and not known to have dementia or mental health illnesses (reported by relative or self-reported), were selected, to give a total of 230 respondents.

### Ethical Statement

The study was approved by the Research and Ethics Committee of the Universiti Kebangsaan Malaysia Medical Centre (UKM 1.5.3.5/244/FF-2013-283). Objectives of the study were explained to all the enrolled respondents, and a written consent was obtained from each respondent. Participants were also assured of confidentiality and they would not be affected by the study outcomes. However patients who scored ‘severe’ and ‘extremely severe’ were informed and advised to seek treatment at nearest health clinic.

### Data Collection and Measures

Data collection was conducted through guided, face to face interviews by trained interviewers, using a structured questionnaire on the information of the respondent’s age, gender, marital status, living arrangement, working status, educational level, personal income, general health status, presence of chronic disease, presence of physical disability, and smoking status. Interviewers were trained by the researchers to ensure the consistency during the interview sessions.

Mental health was assessed using a validated Malay version of the Depression, Anxiety and Stress Scale (DASS-21) [12]. It was seen to have a high internal consistency with Cronbach’s alpha values of 0.84, 0.74, and 0.79, respectively, for depression, anxiety, and stress. The DASS-21 has 21 questions, divided into seven items each assessing the symptoms of depression, anxiety, and stress. For each question, the respondents were asked to rate their experience on each symptom over the past week, on a four-point severity scale, ranging from 0 (does not apply to me), to 3 (applies to me most or all of the time). The scores of every mental health factor were later summed up and categorized as ‘normal’, ‘mild’, ‘moderate’, ‘severe’ or ‘extremely severe’, according to the DASS Manual, with a maximum score of 21 and minimum score of 0 [13]. For this study, the categories were re-grouped into two categories, which were ‘absent’ and ‘present’, for each mental health problem. ‘Absent’ would consists of those with normal mental health (scores of 0 to 4) and ‘present’ would then subcategorized into ‘mild’, ‘moderate’, ‘severe’ or ‘extremely severe’ mental health (scores of 5 and above).

### Statistical Analysis

Descriptive analyses such as frequencies and proportion percentages, mean values and standard deviations, were used to examine samples’ characteristics. Bivariate analyses using Chi-square tests were conducted to explore the difference in depression, anxiety and stress conditions based on samples’ characteristics. Multiple logistic regression analyses were performed using ‘enter’ method to estimate the associations between the dependent (depression, anxiety and stress) and the independent variables (samples’ characteristics) in the study. Only significant variables in bivariate analyses were included in the regression analyses. Missing value data were excluded in the multiple logistic regression analyses. All significant factors were tested for an overall fit of the model using the Hosmer Lemeshow Test, and classification table. Interactions were checked between the independent variables and no interaction was found. Odds ratios (OR) along with 95% confidence levels (CI) were used to quantify the strength of the association. Adjusted odds ratios were presented by controlling other independent variables entered in the multiple logistic regression model. The statistical significance for all tests was set at p<0.05. Statistical analyses were performed using the Statistical Package for the Social Science (SPSS) version 20.0 software.

## Results

### Socio-Demographic Characteristics of the Elderly Respondents

The average age of the respondents was 69.13 (±7.25) years. The oldest respondent among the elderly was 100 years old. The majority of the respondents were female (60.0%), married (66.1%), living with family (69.6%), not working (90.0%), and lower education level (79.1%). There were 84 missing values for the personal income variable. The median of the respondents’ personal income (146 respondents) was RM600 (USD163). Most of the respondents have good health status (69.6%) and absence of physical disability. However 63.5% of them have chronic diseases such as diabetes and hypertension. The characteristics of the respondents, based on socio-demographic status, socio-economic status, general health status, chronic diseases and physical disability status, and smoking status are summarized in Table 1.

**Table 1:** Socio-demographic characteristics of the elderly respondents.

Characteristics of the respondents (n = 230)	n	%
Age (Mean = 69.13±7.25)		
60–74	180	78.3
75 and above	50	21.7
Gender		
Male	92	40.0
Female	138	60.0
Marital status		
Married	152	66.1
Single (Never married, separated, divorcee, widow or widower)	78	33.9
Living arrangement		
Living alone	70	30.4
Living with family (Spouse and/or children)	160	69.6
Working status		
Not working (Unemployed or retired)	207	90.0
Employed	23	10.0
Educational level		
Lower education (No formal education or primary education)	182	79.1
Higher education (Secondary education or tertiary education)	48	20.9
Personal income (Median = RM600/USD163)		
RM1000 and less	108	47.0
More than RM1000	38	16.5
Missing values	84	36.5
Source of income		
Individual (From current job or pension/EPF)	127	55.2
Financial aid (From children, other family members or welfare))	103	44.8
General Health status		
Good	160	69.6
Poor	70	30.4
Presence of chronic disease		
Present	146	63.5
Absent	84	36.5
Presence of physical disability		
Present (Hearing or visual impairment)	35	15.2
Absent	195	84.8
Smoking status		
Smoker	30	13.0
Non-smoker	200	87.0

Abbreviations: EPF = Employees Provident Fund. RM1000 = USD272

### Mental Health Status of the Elderly Respondents

Table 2 shows the mental health status of the respondents based on DASS-21 scores. There were 27.8% having depression, followed by anxiety (22.6%) and stress (8.7%).

**Table 2:** Mental health status of the elderly respondents: scores of Dass-21 subscales.

Mental health status (n = 230)	n	%
Depression		
Present	64	27.8
Absent	166	72.2
Anxiety		
Present	52	22.6
Absent	178	77.4
Stress		
Present	20	8.7
Absent	210	91.3

### Respondents’ Characteristics by Depression, Anxiety and Stress

The respondents’ characteristics that were significantly associated with depression among the respondents were marital status (*p* = 0.010), living arrangement (*p*<0.001), and general health status (*p*<0.001) (Table 3).

**Table 3:** Respondents’ characteristics by depression, anxiety and stress status.

Factor	Depression (n = 230)		p-value	Anxiety (n = 230)		p-value	Stress (n = 230)		p-value
	Present n = 64 (%)	Absent n = 166 (%)		Present n = 52 (%)	Absent n = 178 (%)		Present n = 20 (%)	Absent n = 210 (%)	
Age									
60–74	52 (28.9)	128 (71.1)	0.495	42 (23.3)	138 (76.7)	0.618	15 (8.3)	165 (91.7)	0.711
75 and above	12 (24.0)	38 (76.0)		10 (20.0)	40 (80.0)		5 (10.0)	45 (90.0)	
Gender									
Male	21 (22.8)	71 (77.2)	0.167	21 (22.8)	71 (77.2)	0.949	11 (12.0)	81 (88.0)	0.152
Female	43 (31.2)	95 (68.8)		31 (22.5)	107 (77.5)		9 (6.5)	129 (93.5)	
Marital status									
Married	34 (22.4)	118 (77.6)	0.010*	32 (21.1)	120 (78.9)	0.431	13 (8.6)	139 (91.4)	0.914
Single (Never married, separated, divorcee, widow or widower)	30 (38.5)	48 (61.5)		20 (25.6)	58 (74.4)		7 (9.0)	71 (91.0)	
Living arrangement									
Living alone	8 (11.4)	62 (88.6)	<0.001*	7 (10.0)	63 (90.0)	0.002*	1 (1.4)	69 (98.6)	0.010*
Living with family (Spouse and/or children)	56 (35.0)	104 (65.0)		45 (28.1)	115 (71.9)		19 (11.9)	141 (88.1)	
Educational level									
Lower education (No formal education or primary education)	49 (26.9)	133 (73.1)	0.552	39 (21.4)	143 (78.6)	0.405	17 (9.3)	165 (90.7)	0.773
Higher education (Secondary education or tertiary education)	15 (31.2)	33 (68.8)		13 (27.1)	35 (72.9)		3 (6.2)	45 (93.8)	
Working status									
Not working (unemployed or retired)	58 (28.0)	149 (72.0)	0.844	47 (22.7)	160 (77.3)	0.916	19 (9.2)	188 (90.8)	0.702
Employed	6 (26.1)	17 (73.9)		5 (21.7)	18 (78.3)		1 (4.3)	22 (95.7)	
Personal income									
RM1000 and less	27 (25.0)	81 (75.0)	0.873	25 (23.1)	83 (76.9)	0.946	10 (9.3)	98 (90.7)	1.000
More than RM1000	10 (26.3)	28 (73.7)		9 (23.7)	29 (76.3)		3 (7.9)	35 (92.1)	
Missing values	84 (36.5)			84 (36.5)			84 (36.5)		
Source of income									
Individual (From current job or pension/EPF)	38 (29.9)	89 (70.1)	0.431	31 (24.4)	96 (75.6)	0.468	11 (8.7)	116 (91.3)	0.984
Financial aid (From children, other family members or welfare)	26 (25.2)	77 (74.8)		21 (20.4)	82 (79.6)		9 (8.7)	94 (91.3)	
General Health status									
Good	33 (20.6)	127 (79.4)	<0.001*	30 (18.8)	130 (81.2)	0.034*	12 (7.5)	148 (92.5)	0.331
Poor	31 (44.3)	39 (55.7)		22 (31.4)	48 (68.6)		8 (11.4)	62 (88.6)	
Presence of chronic disease									
Present	47 (32.2)	99 (67.8)	0.051	37 (25.3)	109 (74.7)	0.191	18 (12.3)	128 (87.7)	0.010*
Absent	17 (20.2)	67 (79.8)		15 (17.9)	69 (82.1)		2 (2.4)	82 (97.6)	
Presence of physical disability									
Present (Hearing or visual impairment)	13 (37.1)	22 (62.9)	0.182	13 (37.1)	22 (62.9)	0.026*	7 (20.0)	28 (80.0)	0.018*
Absent	51 (26.2)	144 (73.8)		39 (20.0)	156 (80.0)		13 (6.7)	182 (93.3)	
Smoking status									
Smoker	9 (30.0)	21 (70.0)	0.776	8 (26.7)	22 (73.3)	0.569	3 (10.0)	27 (90.0)	0.731
Non-smoker	55 (27.5)	145 (72.5)		44 (22.0)	156 (78.0)		17 (8.5)	183 (91.5)	

* = significant *p*-value

The elderly characteristics that were significantly associated with anxiety among the respondents were living arrangement (*p* = 0.002), general health status (*p* = 0.034), and presence of physical disability (*p* = 0.026). Whereas for stress, the characteristics that were significantly associated were living arrangement (*p* = 0.010), presence of chronic disease (*p* = 0.010), and presence of physical disability (*p* = 0.018).

### Factors Associated with Depression, Anxiety and Stress of the Respondents

#### Depression

The factors that were significantly associated with depression among the respondents in multiple logistic regression analysis were marital status (*p* = 0.001), living arrangement (*p*<0.001), and general health status (*p* = 0.012). The final model showed that the single elderly (Adjusted OR = 3.27, 95% CI 1.66, 6.44), living with family (Adjusted OR = 4.98, 95% CI 2.05, 12.10), and poor general health status (Adjusted OR = 2.28, 95% CI 1.20, 4.36) were significantly associated with increased odds of having depression (Table 4). The single elderly were three times more likely to have depression compared to the married elderly. The elderly who lived with family were nearly five times more likely to have depression compared to those living alone, while the elderly with poor general health status were two times more likely to have depression compared to the elderly with good general health status.

**Table 4:** Multiple logistic regression analysis of respondents’ characteristics and depression.

Factors	B	SE	Wald	*p*-value	Adj OR	95% CI	
						Lower	Upper
Marital status	1.183	0.346	11.671	0.001*	3.27	1.66	6.44
Living arrangement	1.604	0.454	12.515	<0.001*	4.98	2.05	12.10
General Health status	0.825	0.330	6.242	0.012*	2.28	1.20	4.36
Constant	-2.915	0.467	39.011	<0.001	0.05		

Abbreviations: B = Beta coefficient, SE = Standard Error, CI = Confidence Interval, Adj OR = Adjusted odds ratio,

* = significant *p*-value

Notes: Depression status: normal (0), depression (1); Marital status: married (0), single (1); Living arrangement: living alone (0), living with family (1); General health status: good (0), poor (1); 0 = reference group

#### Anxiety

Living with family was the only significant factor associated with increased odds of having anxiety (Adjusted OR = 2.68, 95% CI 1.09, 6.57) (Table 5). The elderly who lived with family were nearly three times more likely to have anxiety compared to those living alone.

**Table 5:** Multiple logistic regression analysis of respondents’ characteristics and anxiety.

Factors	B	SE	Wald	*p*-value	Adj OR	95% CI	
						Lower	Upper
Living arrangement	0.986	0.458	4.640	0.031*	2.68	1.09	6.57
General Health status	0.519	0.346	2.247	0.134	1.68	0.85	3.31
Presence of physical disability	0.686	0.410	2.797	0.094	1.99	0.89	4.44
Constant	-2.292	0.404	32.218	<0.001	0.10		

Abbreviation:

* = significant *p*-value.

Notes: Anxiety status: normal (0), anxiety (1); Living arrangement: living alone (0), living with family (1); General health status: good (0), poor (1); Physical disability: absence (0), presence (1); 0 = reference group

#### Stress

There was no significant factor associated with stress status among elderly respondents in this study based on multiple logistic regression analysis (Table 6).

**Table 6:** Multiple logistic regression analysis of respondents’ characteristics and stress.

Factors	B	SE	Wald	*p*-value	Adj OR	95% CI	
						Lower	Upper
Living arrangement	1.637	1.078	2.305	0.129	5.14	0.62	42.49
Presence of chronic disease	1.051	0.808	1.693	0.193	2.86	0.59	13.95
Presence of physical disability	0.713	0.533	1.791	0.181	2.04	0.72	5.79
Constant	-4.725	1.106	18.267	<0.001	0.009		

Notes: Stress status: normal (0), stress (1); Living arrangement: living alone (0), living with family (1); Chronic disease: absence (0), presence (1);Physical disability: absence (0), presence (1); 0 = reference group

## Discussion

Estimates of the prevalence of depression, anxiety, and stress in the elderly had a wide range depending on the definition and procedure used. In our study, the prevalence of depression, anxiety, and stress among the Malay elderly, in the rural community, was 27.8%, 22.6% and 8.7% respectively. Depression among the elderly had been widely studied and its prevalence ranged from 6% to as high as 36%, measured in different settings, which included community, out-patient clinics, and old folks’ homes [9–10,14–19]. In Malaysia, studies showed that the prevalence of depression among the elderly in the community ranged from 6.3% to as high as 30.1% [10,14,20]. This present study showed that the prevalence of depression among the Malay elderly was high, which was in concordance with the study by Rashid *et al*. [20], conducted in a similar rural community in northern Malaysia, with a reported prevalence of depression of 30.1%. The prevalence of anxiety among the elderly in the community was found to be almost as high as the prevalence of depression, which ranged from 11 to 21% [21–23]. This present study showed that the prevalence of anxiety was also high among the Malay elderly. Prevalence of stress among the elderly was not well-discussed in literature.

One study by Albright [24] had found that the prevalence of stress among the immigrant elderly in California was 27%, which was three times higher compared to this present study. Nevertheless, it seems difficult to compare the prevalence of anxiety and stress in this study with others, since the study respondents were all Malay, and they are not well-discussed in the Malaysian literatures.

With regards to significant factors associated with depression from multiple logistic regression, in our study there were three factors identified; being single, living with family, and poor general health status. Of the three factors, living with family was found as the only significant factor for anxiety. There was no significant factor found for stress among the Malay elderly in the rural community. On the other hand, marriage has been shown to be a protective factor against depression in the elderly. The finding of this study was similar to other previous studies [15,16,20], which showed higher risks of depression among the single (unmarried, divorced, and widowed) elderly. It was assumed that married life and having a spouse as a life partner provided a feeling of safety, being secured, and a feeling of connectedness that influenced mental health. These are also norms among the rural community, emphasizing on family institution in any communal activities, hence, in the case of being recently single, might lead to a feeling of isolation and loneliness, whichin itself could increase the risk of depression. This assumption is acceptable to anyone, regardless of their age, ethnicity, cultural background or area of living.

Conversely, it was interesting to find that those living with family (either with spouse and/or children) had a higher risk of depression and anxiety, compared to those who lived alone. The living arrangement was not a significant factor for stress among the Malay elderly based on the multiple logistic regression analysis. This finding showed that the living arrangement had left mental health consequences among the Malay elderly in the rural community. However, this finding was not conclusive, as living with a spouse independently or an elderly couple living with their children was categorized together in one group. Previous studies looking into the association between depression and living arrangements were mixed in theirfindings. Some studies had showed similar findings that living with family led to depression [25,26], whereas, several studies demonstrated that living alone had a higher risk of depression compared to living with family [18,20,27]. The heterogeneous nature of the findings could be explained by the different types of living arrangements applied in the studies. United Nations has made a classification of living arrangements which are: (1) Living alone; (2) Living with spouse only; (3) Living with a child, child-in-law or grandchild; (4) Living with another relative (other than a spouse or child/grandchild) and; (5) Living with unrelated people only, apart from the older person’s spouse[28]. It was hypothesized that the elderly were more comfortable having a family around, as there would be more interaction between the elderly and the family members providing social and emotional support, thus improving the mental wellbeing of the elderly. Kooshiar *et al*. [29] supported the observation, in which they concluded that living with the family provided a higher level of a social support system, compared to those living alone. However, the results of our study showed otherwise. Our conjecture to this reverse finding was that the elderly might find living with family members depriving them of the ‘me-time’, either to spend it alone or by participating in social activities in the community. Conforming to the living arrangement of Asian extended families, the elderly are often given the responsibilities of caring for the household, including carrying out the household chores and looking after grandchildren.

Studies have shown that there is a likelihood that caregiving grandparents are more likely to report poorer self-reported health status and are twice more likely to report clinically relevant levels of depressive symptoms [30], compared to the elderly who live alone and are still healthy. These elderly may have more time to involve in social activities (especially religious activities) with lesser chores to do at home. However, looking into our study observation regarding the single elderly, we see they are three times more likely to develop depression, it shows that life satisfaction at times is not measurable only by interpersonal interactions alone, but may involve complex inter-related factors, namely, social needs, community expectations of the elderly, and expectations of the elderly themselves.

In our study, poor general health status was a significant factor for depression among the Malay elderly in the rural community. This was consistent with a meta-analysis study by Chang-Quan *et al*. [31] which had reported that poor self-reported health status appeared to be more strongly associated with depression than the presence of chronic diseases. Physical disability was not a significant factor of mental health problems among the Malay elderly in the rural community based on the results of multiple logistic regression analyses. Bernabei *et al*. [22] had also reported that sensory impairment in older adults could increase their probability of experiencing anxiety.

### Limitations

Purposive sampling used in this study was not a probability sampling and the samples obtained only from two villages and might not represent the elderly population in the rural community. On the other hand, the data were dependent on the respondents’ subjective assessment or self-reported and no objective measures were incorporated to support their responses, thus being subject to possible recall bias.

## Conclusions and Future Research Directions

Mental health problems among the Malay elderly in the rural community were very worrying. The Malay elderly, who were single (unmarried, divorced, and widowed), living with family (with spouse and/or children), and having a poor general health status had a higher risk of depression. Whereas, living with family is the only factor that has an impact on anxiety. However, no significant factor associated with stress among Malay elderly was found in our study. More equity in the health of the elderly should be created or strengthened in order to intensify the opportunity to identify, diagnose, and treat those with mental health problems. Living arrangement was found to be a very important social factor that had influenced depression and anxiety. A qualitative study design, mainly an in-depth interview with the elderly people and their children may be required in future research, to have a better understanding on the living arrangement issue, to reduce the risk of mental health problems among the Malay elderly in the rural community.
